# HCV Coinfection Associated with Slower Disease Progression in HIV-Infected Former Plasma Donors Naïve to ART

**DOI:** 10.1371/journal.pone.0003992

**Published:** 2008-12-22

**Authors:** Xiaoyan Zhang, Jianqing Xu, Hong Peng, Yan Ma, Lifeng Han, Yuhua Ruan, Bing Su, Ning Wang, Yiming Shao

**Affiliations:** 1 State Key Laboratory for Infectious Disease Prevention and Control, National Center for AIDS/STD Control and Prevention, China CDC, Beijing, China; 2 Shanghai Public Health Clinical Center & the Institutes of Biomedical Sciences, Fudan University, Shanghai, China; 3 Anhui Provincial Center for Disease Control and Prevention, Hefei, China; Beijing Institute of Infectious Diseases, China

## Abstract

**Background:**

It remains controversial how HCV coinfection influences the disease progression during HIV-1 infection. This study aims to define the influence of HCV infection on the replication of HIV-1 and the disease progression in HIV-infected former plasma donors (FPDs) naïve to ART.

**Methodology/Principal Findings:**

168 HIV-1-infected FPDs were enrolled into a cohort study from Anhui province in central China, and thereafter monitored at month 3, 9, 15, 21 and 33. Fresh whole blood samples were used for CD4+ T-cell counting. Their plasma samples were collected and stored for quantification of HIV-1 viral loads and for determination of HCV and Toxoplasma. Out of 168 HIV-infected FBDs, 11.9% (20 cases), 80.4% (135 cases) and 7.7% (13 cases) were infected with HIV-1 alone, HIV-1/HCV and HIV/HCV/Toxoplasma, respectively. During the 33-month follow-up, only 35% (7 out of 20 cases) HIV-1 mono-infected subjects remained their CD4+ T-cell counts above 200 cells/µl and retained on the cohort study, which was significantly lower than 56% (75 out of 135 cases) for HIV/HCV group and 69% (9 out of 13 cases) for HIV/HCV/Toxoplasma group (p<0.05). CD4+ T cells in HIV mono infection group were consistently lower than that in HIV/HCV group (p = 0.04, 0.18, 0.03 and 0.04 for baseline, month 9, month 21 and month 33 visit, respectively). In accordance with those observations, HIV viral loads in HIV mono-infection group were consistently higher than that in HIV/HCV group though statistical significances were only reached at baseline (p = 0.04).

**Conclusions/Significance:**

These data indicated HCV coinfection with HIV-1 is associated with the slower disease progression at the very late stage when comparing with HIV-1 mono-infection. The coinfection of Toxoplasma with HIV and HCV did not exert additional influence on the disease progression. It will be highly interesting to further explore the underlying mechanism for this observation in the future.

## Introduction

The co-infection of hepatitis C virus (HCV) and HIV-1 may result in the interaction between the two viruses and thereby alter the disease course. Patients co-infected with HCV and HIV more rapidly progress to cirrhosis than those with HCV alone [1], and are at increased risk of death from the end-stage liver disease [2]. HAART therapy has been reported to reduce intrahepatic HCV loads. Patients treated with regimen containing protease inhibitor (PI) for at least 6 months had a three- to four-fold lower intrahepatic HCV loads than that observed in patients who had never received any ARV treatment of PI containing regimen or had withdrawn for more than 6 months before the liver biopsy [3], however, no difference was observed in the plasma HCV loads among those groups [4]. It remains controversial how HCV infection affects the disease course of HIV-1 infection. In the Swiss Cohort Study HCV coinfection was independently associated with a 21% reduction in the likelihood of increasing the CD4+ T-cell counts by at least 50 cells/µL and with an increased risk of progression to AIDS and to death among patients initiating HAART [5], and higher HCV RNA levels were associated with more CD4+ T-cell depletion in a prolonged observation (4 years) [6]. In the Johns Hopkins cohort, no significant difference was observed between HIV-1 mono-infection and HIV plus HCV co-infection after controlling for the use and effectiveness of HAART [7]. Interestingly, one study observed that immune recovery is associated with a persistent increase in plasma HCV RNA, especially for those with baseline CD4+ T-cell counts <350 cells/mm, and HCV co-infection did not antagonize the CD4+ T-cell response to HAART [8]. Currently, HIV-1 and HCV co-infection is gradually being recognized as a separated entity from HIV-1 or HCV mono-infections with an altered response to HAART and requires special effort for care and treatment.

In China, HCV co-infection is frequently observed among HIV infected patients, especial in patients of injecting drug users and former plasma donors (FPDs) [9]. Unregulated commercial plasma collection among farmers occurred between 1992 and 1995 in central China, including Henan, Anhui and Shanxi provinces, and caused the second major epidemic of HIV-1 infection in China. Although this was eradicated by Chinese government by the end of 1995 [10], the practice of using contaminated blood collection equipment or re-infusing pooled blood cells back to donors caused rapid HIV-1 and HCV spreading among those FPDs [11]–[12]. HIV-1-infected FPDs represent a unique population to study the natural disease progression of HIV-1 and HCV coinfections because the outbreak of infection occurred within a narrowed period and the vast majority of FPDs were infected with HIV-1 and HCV from a common-source exposure to contaminated blood and currently have had a >10 years infection history, any manifestation from HIV-1 on HCV or from HCV on HIV-1 should have been accumulated for more than a decade for observation.

We established an ART naïve cohort of HIV-1 and HCV co-infection in FPDs and a 33-month longitudinal observation has been carried out. In addition, to exclude the interference from other pathogens, we further determined the influences of the infections toxoplasma. Our observation will bring new insight to the management of HIV/AIDS patients to reduce their morbidity and mortality.

## Materials and Methods

### Establishment of study cohort

HIV-1-infected former plasma donors (FPDs) naïve to anti-retroviral therapy in Fuyang prefecture city of Anhui Province in central China were enrolled into this study. All participants donated blood between 1992 and 1995 from their self-report and HIV+ individuals in the absence of blood donation history during that period were excluded from our study cohort. Since this is a conservative area and no other high-risk behaviors (such as drug using or extramarital sexual contacts) were reported in this cohort, their HIV infection were epidemiologically attributed to a common-source exposure to contaminated blood during their practice of blood donation,which included using contaminated blood collection equipment or re-infusing pooled blood cells back to donors. Following approval by IRBs at China National Center for AIDS/STD Control and Prevention and at Anhui Provincial Center for Disease Control and Prevention, 294 HIV-1-infected FBD volunteers naïve to anti-HIV and anti-HCV treatment were screened in June, 2005 [13], including 77 cases with CD4+ T cells <200 cells/ul and 217 cases with CD4+ T cells >200 cells/µl. Out of 217 subjects with CD4+ T cells >200 cells/µl, 168 subjects were willing to participate into a Cohort Study in September, 2005, and have been successfully followed up either for 33 months or until CD4+ T cells dropped below 200 cells/µl. All study participants were coded and delinked from their personal information in this study. Subjects whose CD4+ T cells dropped below 200 cells/µl during our follow-up visit were referred to China CARES program. The China CARES program is a comprehensive community-based HIV/AIDS treatment, care, and prevention program. It was launched in early 2003, and has been started in seven provinces in Central China, including Anhui Province. The activities of this program include advocacy, communication, preventive services, voluntary counseling and testing, free treatment of opportunistic infections, free antiretroviral therapy with the regiment D4T+3TC+NVP, and community-based care and support.

### Samples collection

Whole blood specimens were collected in sterile tubes using EDTA as the anticoagulant. All samples were transferred to laboratory within 12 hours after collection. Fresh whole blood samples were used for CD4+ T-cell counting; and plasma samples were stored at −20°C for HIV, HCV and taxoplasma testing. Plasma samples were stored at −80°C for viral RNA extraction and viral load detection.

### Plasma Testing

HIV and HCV antibodies were tested by ELISA (Beijing Wantai, Beijing, China). Toxoplasma Enzyme-linked immunosorbent assay (ELISA) kitswas from Haitai Biocompany (Guangdong, China), the test determined the presence or absence of anti-T. gondii immunoglobulin G (IgG), IgM antibodies. Serum samples were assayed in duplicate. All assays were carried out according to the manufacture's instruction.

### Quantitation of plasma HIV-1 viral loads

Viral RNA was isolated from 200 µl EDTA anticoagulated plasma using a QIAamp Viral RNA kit (Qiagen, Germany) and treated with DNase I on a spin column (DNase 1 set; Qiagen) according to the manufacturer's instructions. RNA was eluted in 60 µl AVE buffer and analyzed immediately. Real-time RT-PCR was used to determine plasma viral load by amplification of a 102-bp segment of the HIV-1 gag gene. The reaction composing and condition were the same as the Manufacture's Manual. Briefly, Real-time RT-PCR was performed using Real-time RT-PCR Kit (**S**henzhen PG Biotech Co. China) in 10 µl reaction volume. Cycling conditions were 30 min at 42°C, 3 min at 92°C, followed by 5 cycles at 95°C for 10 s and 52°C for 20 s, and 72°C for 30 s., then another 40 cycles at 95°C for 5 s and 60°C for 30 s, and 40°C for 30 s. Reactions were performed in triplicate using a COBAS AMPLICOR Analyzer. Results are expressed as HIV-1 RNA copies/ ml. The low limitation was 500 copies /ml.

### Quantification of CD4+ T-Cell Counts

CD4+ T lymphocyte absolute counts can be accomplished with single platform counting technologies employing cytometric methods. All reagents were from Becton Dickinson (CA). Twenty microliter antibodies (CD3-FITC/CD4-PE/CD45- PerCP) were added into different TruCOUNT tubes, then 50 µl whole blood was added into each TruCOUNT tube and mixtures were gently vortexed at room temperature (18°C–25°C) in the dark for 20 minutes. Next, 450 µl 1×FACS lysing solution (Dilute 10×FACS lysing solution with distilled water) was added into each tube. The mixtures were incubated at 20°C–25°C in the dark for 15 minutes, then run within 24 hours.

### Statistical analysis

The Student's *t* test was used to compare the HIV-1 viral load and CD4+ T cells counts among different groups. Statistical significance was defined as a p value of <0.05.

## Results

### Characterization of the study cohort

It was estimated that about 1400 subjects were infected by HIV-1 through the blood/plasma donation in Fuyang city of Anhui province during 1992–1995 and ∼50% of them (∼700 subjects) have died from AIDS after more than a decade of HIV-1 infection (data from local CDC report). Among the left 700 subjects (∼50%) survivals, >400 of them are receiving free ART, thus our cohort (168 subjects) represents ∼60% ART-free subjects in this region. The demographic and epidemiological data of this cohort were shown in Table 1. The high-risk factors for acquired HIV-1 infection were all from unregulated blood/plasma donation. According to our previous study, HIV-1 strains prevalent in this cohort were absolutely subtype B′, no viral property was identified to be associated with low CD4+ T-cell counts and only two possible paired transmission may occurred in the past according to *env* gene sequencial analysis [14]. In addition, HCV 1b and 2a subtypes dominated in FPDs population [9], [15]. Out of the 168 PBDs, female subjects accounted for 43.1% and male subjects accounted for 56.9%, and the average age was 44.27±8.13.

**Table 1:** Characterization of study cohorts.

Category	Subcategory	HIV-1 monoinfection (N = 20)	HIV/HCV dual infection (N = 135)	HIV/HCV/ Toxoplasma triple infection (N = 13)	Total (N = 168)
High-risk	Blood/plasma donation	Yes	Yes	Yes	Yes
	Drug using	No	No	No	No
Age (Mean±SD)		46.3±11.1	44±7.8	42.5±5.5	44.27±8.13
Sex	% Female (cases)	50 (10)	41.5% (56)	69.2% (9)	43.1% (75)
	% Male (cases)	50 (10)	58.5% (79)	30.8% (4)	56.9% (99)
HIV-1 subtype		B′	B′	B′	B′

### HCV coinfection with HIV-1 associated with higher CD4+ T cell counts and lowering HIV viral loads at baseline

**Figure 1:**
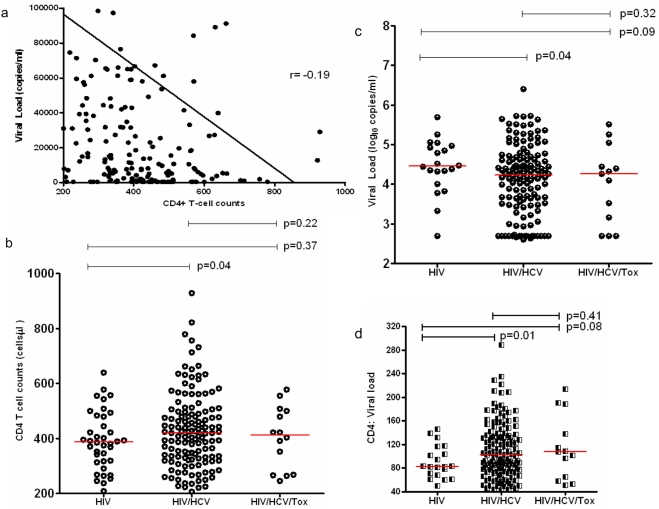
Characterization of the cohort at baseline. a, the reverse association of CD4+ T-cell counts and viral loads in the study subjects at baseline. The X axis indicated CD4+ T-cell counts (cells/µl), and Y axis indicated viral loads; GraphPad Prism 5 was used to generate the trend line and r value. b and c, CD4+ T-cell counts and viral load among different groups at baseline. The X axis indicated different groups including HIV (HIV-1 mono infection), HIV/HCV (dual infection), and HIV/HCV/Tox (triple infection of HIV, HCV and toxoplasma). The Y axis indicated CD4+ T-cell counts (1b) and viral load (1c); d, the ratio of CD4+ T cells to viral loads among different groups, the ratios were calculated by dividing CD4+ T-cell counts by the value of viral loads in log10.

The overall CD4+ T-cell counts were negatively associated with plasma viral loads (r = −0.19) at baseline (Fig. 1a), and this negative association were consistently observed during the follow-up visits (data not shown), indicating that the viral replication is the driving force for disease progression in the FBDs population. To determine the effect of HCV coinfection with HIV-1 on the disease progression, we compared HIV viral loads and CD4+ T-cell counts at baseline among HIV-1 mono-infection (N = 20 cases), dual infection of HIV plus HCV (N = 135 cases) and triple infection of HIV, HCV and toxoplasma (N = 13 cases). The reason to include the toxoplasma is to test whether the toxoplasma coinfection with HIV and HCV could exact any additional effect on disease progression.

As shown in figure 1b, CD4+ T-cell counts in the group of dual infection (ranged from 206 to 928 cells/µl with a mean of 429 cells/ µl) was significantly higher than that in HIV mono-infection (ranged from 237 to 639 cells/ul with a mean of 390 cells/µl) (p = 0.04). Similarly, CD4+ T-cell counts in the group of triple infection (ranged from 245 to 577 cells/ul with a mean of 404 cells/ µl) also tended to be higher than that in HIV mono-infection though statistical significance was not reached. In contrast, HIV-1 viral loads in the group of HIV/HCV dual infection (ranged from under detection threshold to 5.77 with a mean of 4.15 log_10_ copies/ml plasma) were significantly lower than that in the group of HIV mono-infection (ranged from under detection threshold to 5.24 with a mean of 4.43 log_10_ copies/ml plasma) (p = 0.04), the difference was further expanded between the triple infection of HIV/HCV/Toxoplasma (ranged from 2.63 to 5.19 with a mean of 4.03 log_10_ copies/ml plasma) and HIV-1 mono-infection, however, no significant difference was observed between the dual infection and triple infection groups (p = 0.14) (Fig. 1c). Since CD4+ T-cell count is the restraining factor and viral loads is the driving force of disease progression, the ratio of CD4+ T cells to viral loads will be likely a useful predictor for disease progression and the lower ratio will be associated with faster disease progression. Therefore, we examined the ratios of CD4+ T cells to viral loads in log10 in our cohort. The ratio in the group of HIV/HCV dual infection (ranged from 43.3 to 288.8 with a mean of 110.6) were significantly higher than that in the group of HIV mono-infection (ranged from 49.6 to 145.5 with a mean of 90.5) (p = 0.01), a similar difference was also observed between triple infection (ranged from 51 to 213.8 with a mean of 114.3) and mono-infection though statistical significance was not reached (p = 0.08) (Fig. 1d). These data indicate HCV coinfection with HIV-1 may slow down disease progression of HIV-1 infection and toxoplasma exerts highly limited effect on disease progression.

### HCV coinfection with HIV-1 associated with slower disease progression in cohort study

**Figure 2:**
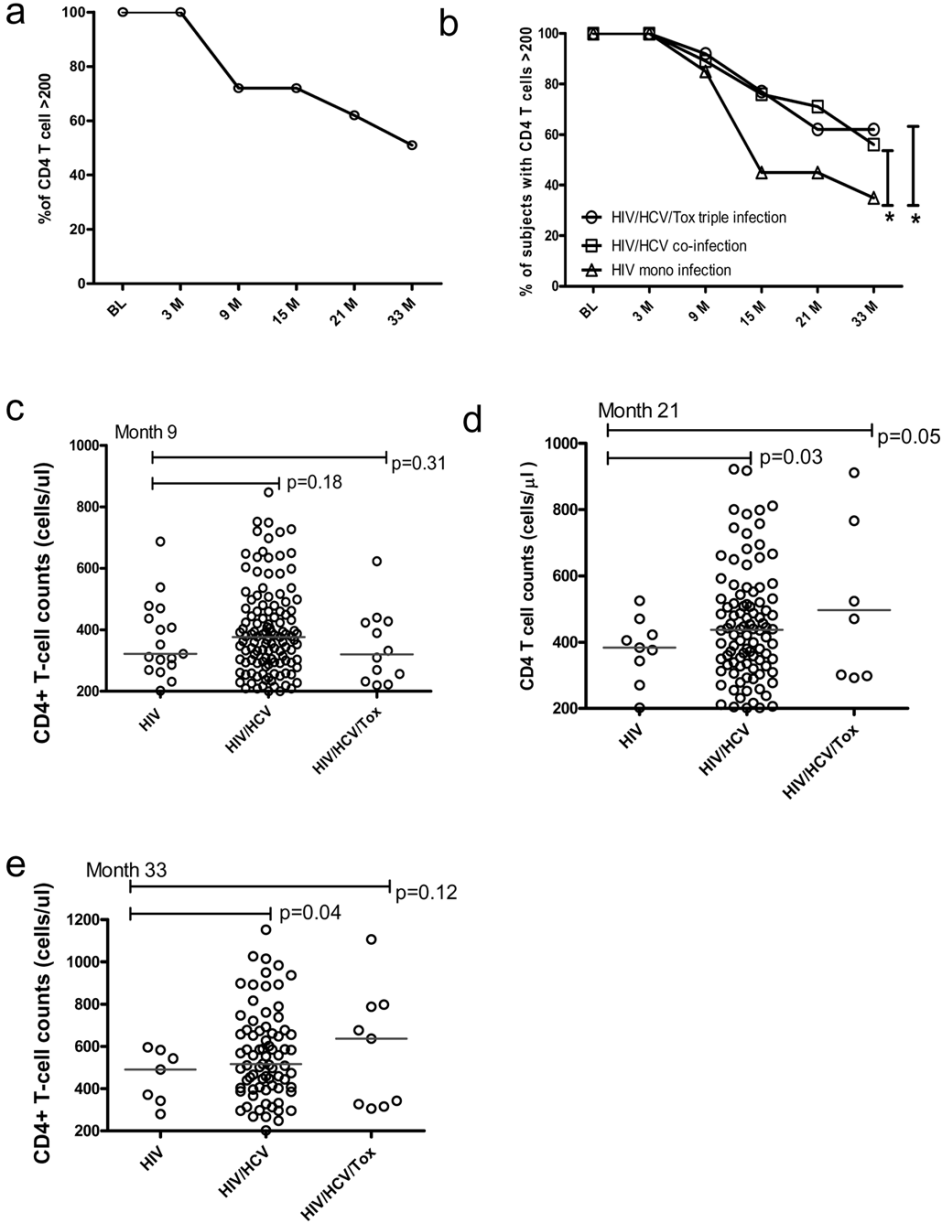
Disease progression during our follow-up. a, the percentage of subjects remained with CD4+ T cell counts >200 cells/µl during the follow-up visits The X axis indicates the different time-points of sampling at baseline (BL), month 3 (3M), month 9 (9M), month 15 (15M), month 21 (21M), month 33 (33M) after enrollment; Y axis is the percentage of subjects whose CD4+ T-cell counts remained as >200; b, the percentage of subjects remained with CD4+ T-cell counts >200 cells/µl during the follow-up visits among different groups as indicates in the figure, X and Y axis are labeled identically to figure 2a;. * indicated p<0.05; c, d, e, CD4+ T-cell counts among different groups at month 9 (c), month 21 (d) and month 33 (e).

**Figure 3:**
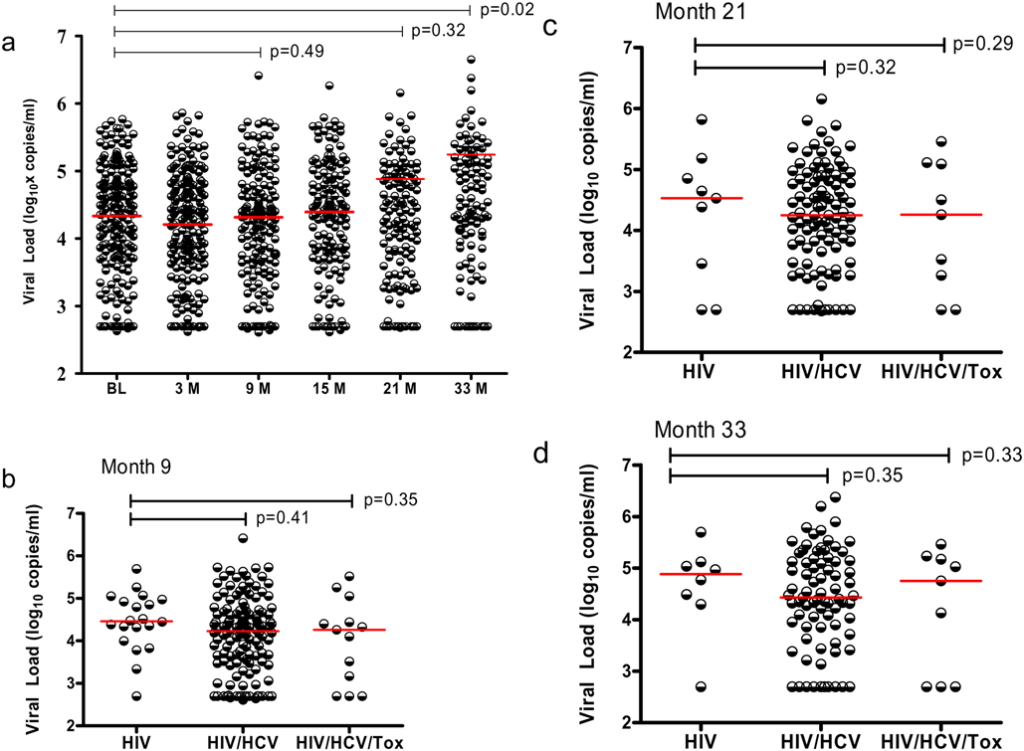
Viral loads at different follow-up visits (a) and among different groups (b, c, d) during observation. The X axis indicates the different time-points of sampling at baseline (BL), month 3 (3M), month 9 (9M), month 15 (15M), month 21 (21M), month 33 (33M) after enrollment (a) or indicates different groups (b, c and d), and Y axis indicates the viral load (log_10_ copies/ml).

To further determine the influence of HCV coinfection with HIV-1 on disease progression, we conducted a prospective cohort study and calculated the percentages of individuals whose CD4+ T-cell counts remained >200 cells/µl and retained on the cohort study. During 33 month follow up, the percentage of subjects with CD4+ T-cell counts >200 cells/µl in this cohort decreased at month 9 visit after entry (p<0.05) and continued to drop afterwards (p<0.01 for all other visits comparing to baseline data). At the final time point (33-month), only 51% of study subjects were remained with CD4+T cells over 200 cells/µl (Fig. 2a). We then examined the retaining rate by groups. As shown in figure 2b, no significant differences of the percentages among groups were observed during the first 9-month observation though the difference did appear at month 9 visit. These data were also supported by the observation from the baseline, 25.8% HIV-1 mono-infection cases (8 out 31 cases) had CD4+ T cells below 200 cells/µl, which is comparable to what observed in HIV/HCV dual infection group (65 out of 253 cases, 25.7%). However, during the prolonged observation (at month 15, month 21 and month 33 visits), significant less fractions of subjects from HIV/HCV dual infection or HIV/HCV/Toxoplasma triple infection progressed into AIDS stage defined as CD4+ T cells below 200 cells/µl (Fig. 2b). At the month 33 follow-up, only 35% HIV-1 mono-infected cases remained their CD4+ T-cell counts >200 cells/µl, which is significantly lower than that observed 56% in HIV/HCV dual infection group and 69% in HIV/HCV/Toxoplasma triple infection group. Furthermore, absolute CD4+ T-cell counts in HIV/HCV group were consistently higher than that in HIV mono-infection group (p = 0.04, 0.18, 0.03 and 0.04 for baseline, month 9, month 21 and month 33 visit, respectively). 20% (27 cases) retained their CD4+ T-cell counts >600 in HIV/HCV group even at month 33 visit whereas none does so in HIV mono-infection group since month 21 visit (Fig. 2c, d and e). In accordance with those observations above, HIV viral loads in HIV/HCV group were consistently lower than that in HIV-1 group though statistical significances were only reached at baseline (p = 0.04) (Fig. 1c) and not other visits (Fig. 3). These data indicated that HCV coinfection with HIV-1 may significantly slow down the disease progression and this effect may only manifest at the very late stage of HIV-1 infection.

### HCV coinfection with HIV-1 may associate with slower disease progression in the future in this cohort

**Figure 4:**
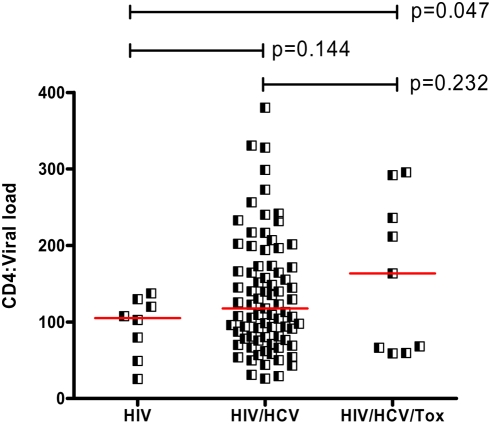
Ratio of CD4+ T-cell counts to viral loads in log10 among different groups at month 33 visit. X axis indicates different groups, and Y axis indicates the ratio values of CD4+ T-cell counts (cells/µl) to viral load (log_10_ copies/ml).

As described above, the ratio of CD4+ T cells to viral loads may be a useful predictor for disease progression of HIV-1 infection, our baseline data (Fig. 1d) did correlate with the disease progression data from the cohort study, the low ratio in HIV-1 mono-infection did progress faster than HIV/HCV group with higher ratio. We thus further determined the ratio of CD4+ T cells to viral loads at month 33 visit (Fig. 4). The ratio for HIV-1 mono-infection group ranged from 25.5 to 137.5 with a mean of 94, which was significantly lower than that in HIV/HCV dual infection group (ranged from 26.1 to 380.1 with a mean of 134.7) (p = 0.014) and in HIV/HCV/Tox triple infection group (ranged from 59 to 295.6 with a mean of 161.3) (p = 0.046). We speculate that HCV coinfection with HIV-1 may still significantly slow down the disease progression in the cohort in the future.

## Discussion

Though HIV-1 co-infection with HCV has been recognized to facilitate the HCV replication and liver disease progression [16], it remains to be determined on the role of HCV in the disease course of HIV infection. A large cohort study of HIV-1-infected subjects has demonstrated that HCV co-infection with HIV-1 accelerated the progression to a new AIDS-defining clinical event or to death through altering the immunological but not virological responses to HAART [5]. Several small cohort studies observed that a faster disease progression was coincident with HCV coinfection with HIV-1 [17]–[20]. However, the association between HCV coinfection and AIDS-related disease progression was not defined in EuroSIDA cohort study and several other studies [21]–[23]. Furthermore, HCV coinfection was correlated with long-term non-progression status [24]. The controversial observations in this field were best exemplified by two pairs of conflicting cohort studies. The first is that a small cohort study for following up10 years in children defined that the HCV seropositive children had twofold higher risk of progression to the development of AIDS than HCV seronegative individual [25], whereas another study in children who were vertically infected unambiguously demonstrated that significant less HIV-1/HCV coinfected children developed AIDS than HIV-1 infected alone during a long-term observation [26]. The second is that one study showed patients coinfected with HCV genotype 1 had a faster rate of AIDS-related disease progression [27], and another demonstrated that HCV genotype 1 was associated with long-term non-progression [28].

We took the advantage of HIV-1 infected FPDs cohort naïve to ART for more than a decade and longitudinally monitored the disease progression for almost three years. HIV-1-infected FPDs represent a unique population to study the natural disease progression of HIV-1 and HCV coinfections because the outbreak of infection occurred within a narrowed period and the vast majority of FPDs were infected with HIV-1 and HCV from a common-source exposure to contaminated blood and currently have had a >10 years infection history. Thus, this cohort are composed of long-term non-progressors and late progressors with epidemiological data support of a similar virological background and a narrow-windowed time of initial infection, we believed that these properties render our cohort is highly suitable for defining the influence of HCV on HIV-1 infection. Our data demonstrated that patients coinfected with HCV and HIV-1 was associated with significant higher CD4+ T-cell counts than that in HIV-1 mono-infection group, furthermore, co-infected individuals also tended to have lower viral loads. Importantly, significant less percentage of individuals in HIV/HCV coinfected group progressed into AIDS stage defined as CD4+ T-cell counts <200 **cells/µl** during our observation. Finally, the HIV/HCV group has significant higher ratios of CD4+ T cells to viral loads than that in HIV-1 mono-infection even at month 33 visit, which may indicate that the HIV/HCV group will be likely to progress slower than the HIV-1 mono-infection group in the future. Since our previous virological study demonstrated that all participants were infected by Thailand B clade, no viral property was identified to be associated with disease progression and only two possible paired transmission may occurred long time ago according to *env* gene sequencial analysis [14], thus, the observed difference between HIV-1 monoinfection and HIV/HCV dual infection was highly unlikely to be explained by different HIV-1 viral characterization. Therefore, our data support previous reports in long-term non-progressors and in a children cohort [26]. As previously reported that the FBDs were infected by HCV 1b and 2a [9], [15], it remains unknown whether those particular genotypes could contribute to the lower viral loads and slower disease progression observed in this cohort.

As know, toxoplasma infection may speed up the AIDS-related disease progression, particularly in those with their CD4+ T-cell counts below 200 cells/µL [29]–[30]. Though we excluded patients with CD4+ T-cell counts <200 cells/µL at baseline from our cohort study, however, toxoplasma may exert influence during our follow-up visits when participants' CD4+ T-cell counts dropped, therefore, we performed toxoplasma testing and excluded toxoplasma infected individual from the HIV-1 monoinfection. To test whether toxoplasma infection could influence the HCV/HIV-1 group, we compared the viral loads and CD4+ T-cell counts between HIV-1/HCV dual infection group and HIV-1/HCV/Toxoplasma triple infection, our data showed that toxoplasma infection did not play a significant role in the viral replication and disease progression.

Though it remains to be defined for the observed discrepancies between our data and several previous reports, several factors should be taken into consideration. The cohorts monitored by previous studies might represent a mixture of patients at different progression stages, our cohort represents a relative pure population at very late stage. We hypothesized that HCV coinfection may exert varied influences among different HIV-1-infected individuals, patients with negative influences from HCV coinfection should have had a faster disease progression and may dominate the observed results in cohort studies on patients at early stage or on a mixture of patients at different stages since only a small fraction of individuals will survive for a long term, whereas patients with positive influences from HCV infection will gradually become predominant in late stage. In accordance with this hypothesis, our data showed that though CD4+ T cells counts in HIV/HCV dual infection group were significantly higher and viral loads were significantly lower than that in HIV-1 monoinfection group at the baseline, a similar fraction of individuals in both groups was observed at AIDS stage with CD4+ T cells <200 cells/ul; it was only appeared at the last three visits that a significant less fraction of HIV/HCV dual infected subjects progressed into AIDS. A recent report also supported our hypothesis, HCV coinfection with HIV-1 generated two distinct patterns of immune responses, some patients had a limited response to either virus whilst others made responses to a range of HIV epitopes, interestingly, HCV responses were detected only in those who made multiple responses to HIV epitopes. Lack of HIV-specific T cells was associated with a decline in absolute CD4+ T-cell counts between the time points [31]. These data indicated that the host immune system either responded to both viruses or to neither viruses during coinfection, the non-responded individuals may reflect the faster progression group. Further cohort study at very early stage will be required to validate this concept.

Overall, our data demonstrated that HCV coinfection with HIV-1 was associated with slower disease progression in HIV-1 infected former plasma donors naive to ART at the very late stage.
